# Supplementary material for: Isolation and Identification of Aeromonas veronii in Sheep with Fatal Infection in China: A Case Report
Source: Microorganisms. 2023 Jan 29;11(2):333. doi: 10.3390/microorganisms11020333 (PMC9961254; doi:10.3390/microorganisms11020333)
Supplement: Supplementary file 1 [file microorganisms-11-00333-s001.zip › microorganisms-2126351-supplementary.pdf]

Supplementary Materials:

Table S1 Description of sick and dead sheep affected by this outbreak

		sick	dead
Rate		2.53% (15/594)	40.0% (6/15)
Gender	Male	6/15	1/6
	Female	9/15	5/6
Age (month)	<6	8/15	3/6
	6-12	5/15	1/6
	>12	2/15	2/6
Clinical signs		dyspnoea, nasal discharge, wet cough, hyperthermia	dyspnoea, nasal discharge, diarrhea, wet cough, emaciation
Treatment		ampicillin, tylosin, florfenicol, ceftiofur	
Lab analysis (YES or NO)		YES	YES

Table S2 Primers used in this case report

Primer sequence (5'-3')		pathogen	Target gene	Reference
Foward	Reverse			
TGAACGGAATATGTTAGCTT	GACTTCATCCTGCACTCTGT	M. O.	16S	[12]
GGACCATACAGGAGAAGTTGA	CGCAGGTAAAGTGATCTGTAGC	FMDV	3D	[13]
ATTGTCCACTATTGAATCCTTGAT	TTGTCGTTGTAGACCTGACTGTTG	PPRV	N	[13]
TGACACAGCAAATGTAACCGCAAG-	CCACGTTGGGCGCCAGCTGCGCGA	MAEDI	gag	[14]
AGTGATCTAGATGATGATCCA	GTTATTGATCCAATTGCTGT	PIV	M	[15]
TGCCAAAACATAACAGAAG	TTTATTCACCTCTCCCTC	RSV	F	[16]

Table S3 Biochemical characteristic of strain YL4077

Test	Result	Test	Result
Peptone	+	Sorbitol	-
Simmons citrate	+	Voges-Proskauer test	+
Uera	-	H2S	-
Catalase	+	Glucose (gas)	+
Esculin	-	Indole	+
Sucrose	-	Lysine	+
Arabinose	-	Phenylalanine	-
Xylose	-	Ornithine	-
Lactose	+	Nitrate reduction	+
D-mannose	+	Glycerin	+

Note: "+": Positive reaction; "-" Negative reaction.
